# Waist-to-hip ratio and nonalcoholic fatty liver disease: a clinical observational and Mendelian randomization analysis

**DOI:** 10.3389/fnut.2024.1426749

**Published:** 2024-11-01

**Authors:** Weining Xie, Yan Hong, Xinrong Chen, Shujuan Wang, Fan Zhang, Xiaoling Chi

**Affiliations:** ^1^The Second Clinical College of Guangzhou University of Chinese Medicine, Guangzhou, Guangdong Province, China; ^2^Infectious Disease Department, Guangdong Provincial Hospital of Integrated Traditional Chinese and Western Medicine, Foshan, Guangdong Province, China; ^3^Affiliated Guangdong Hospital of Integrated Traditional Chinese and Western Medicine of Guangzhou University of Chinese Medicine, Guangzhou University of Chinese Medicine, Foshan, Guangdong Province, China; ^4^The First Clinical Medical College, Guangzhou University of Chinese Medicine, Guangzhou, Guangdong Province, China; ^5^Department of Hepatology, Guangdong Province Hospital of Traditional Chinese Medicine, Guangzhou, Guangdong Province, China

**Keywords:** non-alcoholic fatty liver disease, waist-to-hip ratio, Mendelian randomization analysis, NHANES, clinical observational study

## Abstract

**Background:**

Obesity often coincides with non-alcoholic fatty liver disease (NAFLD), yet a significant portion of NAFLD patients exhibit normal body mass index (BMI) but have abdominal obesity. Recognizing this discrepancy, we aimed to delve deeper into this phenomenon through observational studies coupled with two-sample Mendelian randomization (MR) analysis, with waist-to-hip ratio (WHR) serving as the indicator for abdominal obesity. Our objective was to ascertain whether WHR correlates with an increased risk of NAFLD development.

**Methods:**

This study utilized data from the National Health and Nutrition Examination Survey (NHANES) 2017–2018 to examine the association between WHR and NAFLD through weighted multivariate logistic regression models. On this basis, subgroup analyses were performed to further explore the correlation between WHR and NAFLD. Subsequently, a two-sample MR analysis was conducted using genome-wide association studies (GWAS) data to investigate the potential causal relationship between WHR and NAFLD. Sensitivity analyses were also employed to ensure the robustness of our findings.

**Results:**

A total of 3,732 eligible participants were included in the analysis. Weighted multivariable-adjusted logistic regression models revealed a positive association between WHR and the risk of NAFLD (Q2vsQ1: OR = 1.94 [95% CI: 1.55–2.44]; Q3vsQ1: OR = 2.08 [95% CI: 1.51–2.85]; Q4vsQ1: OR = 3.70 [95% CI: 2.13–6.43], *p* < 0.05). The results of the subgroup analysis suggested that there was an interaction in the correlation between WHR and NAFLD in normal weight, overweight, and obese populations (*p* < 0.05). The RCS curves indicated that there was a nonlinear relationship between WHR and NAFLD in populations with BMI in the normal versus obese categories. Furthermore, MR analysis provided additional support for the causal relationship between WHR and NAFLD. Using inverse variance weighting (IVW), the MR analysis yielded an OR of 2.062 (95% CI: 1.680–2.531, *p*<0.05). Consistent results were obtained with the other four MR methods, all supporting the same direction of causality. Sensitivity analyses were performed to assess the robustness of the findings (*p* > 0.5), further reinforcing the reliability of the observed associations.

**Conclusion:**

WHR elevation heightens the susceptibility to NAFLD.

## Introduction

Non-alcoholic fatty liver disease (NAFLD) has a global prevalence of 30% (1) and is currently the fastest-growing cause of liver-related deaths worldwide (2). Given that NAFLD is a metabolic disease, it has also recently been designated as Metabolic-Associated Fatty Liver Disease (MAFLD) (3). However, current clinical trials are conducted based on the pathogenesis of NAFLD (4). MAFLD largely depends on associated comorbidities, and the current definition of MAFLD only encompasses 81.59% of NAFLD patients, putting patients with hepatic steatosis without clear clinical metabolic risk factors at a disadvantage (5). Lean NAFLD accounts for as much as 19.20% (6), with a lower likelihood of metabolic dysregulation, making it challenging to diagnose as MAFLD. Therefore, this study continues to focus on NAFLD as the subject of investigation.

NAFLD is a metabolic stress liver injury caused by abnormal accumulation of lipids in hepatocytes and is gradually replacing viral hepatitis as the most common chronic liver disease (7). Without timely intervention and treatment, the disease can progress from simple steatosis (NAFL) to nonalcoholic steatohepatitis (NASH) and even to irreversible cirrhosis and hepatocellular carcinoma (HCC). NAFLD is also closely related to the development of cardiovascular diseases, diabetic complications, chronic kidney disease, and malignancy (8–10). Therefore, it is of utmost importance to prevent and treat NAFLD as early as possible to reduce its progression to adverse outcomes.

Obesity is one of the independent risk factors for NAFLD. A meta-analysis demonstrated that obese individuals have a 3.5-fold increased risk of NAFLD, and there is a significant dose-dependent relationship between obesity and NAFLD risk (11). A variety of human obesity measures have been employed to assess the degree of risk for metabolic diseases in humans, including BMI, waist circumference (WC), waist-to-height ratio (WHtR), and other anthropometric measures. The current common metrics for determining obesity are the BMI. A study by Younossi ZM (12). demonstrated that the prevalence of NAFLD increased with BMI. Nevertheless, an increasing number of studies have demonstrated that NAFLD can also occur in individuals with a normal BMI and an excessive abdominal circumference, or what is commonly referred to as “thin individuals with NAFLD.” A meta-analysis conducted by Tang (13) revealed that thin individuals with NAFLD accounted for approximately 13.11% of the global population, with a prevalence as high as 14.55% in the Asian region. This indicates that the use of BMI alone to diagnose obesity is erroneous. One possible explanation for this is that BMI does not consider the distribution of adipose tissue, particularly in the trunk region (14).

A considerable number of studies have predicted obesity by WHR and used it to identify individuals with abdominal obesity. Furthermore, the correlation between this ratio and NAFLD has been confirmed in numerous studies (15). Golabi (16) have demonstrated that individuals with abdominal obesity are at a greater risk of developing NAFLD than those with general obesity. Furthermore, they have shown that abdominal obesity is associated with an increased risk of all-cause mortality and cardiovascular mortality. These findings suggest that the WHR may be a more accurate indicator of obesity than the BMI.

Nevertheless, abdominal obesity and NAFLD frequently co-occur in the same individual, forming a complex, bidirectional relationship. Existing studies have various limitations, including insufficient sample sizes and cross-sectional designs that are susceptible to confounding factors, making it challenging to ascertain causality. This study utilized high-quality, nationally representative data from the NHANES, a large-scale ongoing study conducted by the National Center for Health Statistics (NCHS). On this data, a cross-sectional study was conducted to analyze the correlation between WHR and NAFLD.

MR has emerged as a valuable complement to cross-sectional observational studies in assessing the causal effect of a specified risk factor on an outcome at the genetic level (17). The advantage of MR is that genetic variants are established before birth and randomly assigned at meiosis and fertilization, which greatly reduces the influence of other factors on the outcome and addresses the issue of avoiding the association between exposure and reverse causation of the outcome (18). Consequently, we employed two-sample MR analysis to supplement cross-sectional studies and comprehensively evaluate the relationship.

## Methods

### Study population

All data utilized in this study were sourced from the National Health and Nutrition Examination Survey (NHANES) database,^1^ a research program designed to assess the health and nutritional status of both adults and children in the United States. The study was authorized by the NCHS Ethical Review Board, informed consent was obtained from all participants, and no additional application for Institutional Review Board approval was required.

**Figure 1:**
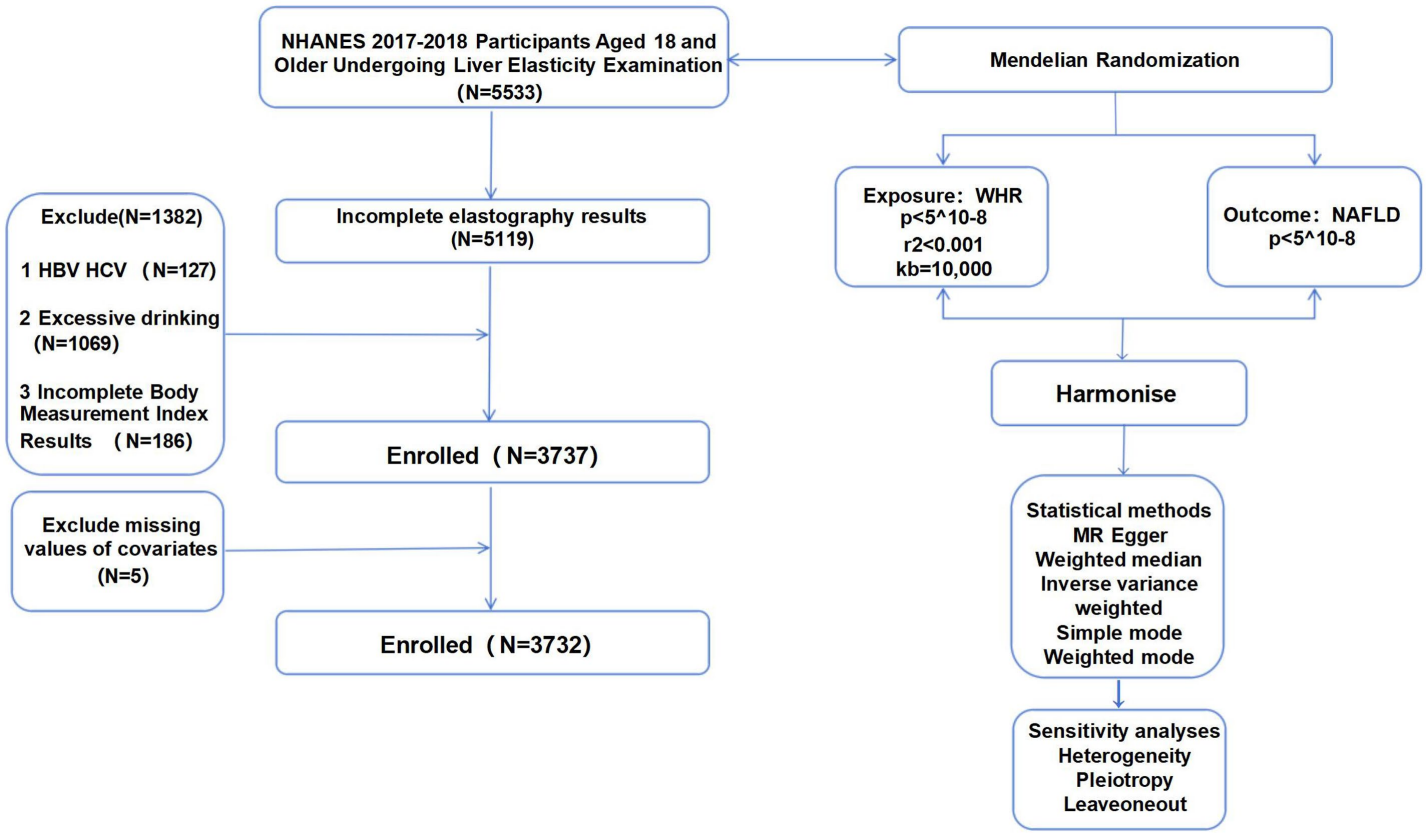
Flowchart.

The research sample consisted of 5,533 individuals who were screened at the NHANES Mobile Screening Center during 2017–2018. These participants were adults aged 18 years and older who provided consent for the FibroScan® Touch to be used for elastography measures. Exclusion criteria included: (1) incomplete elastography results (*N* = 414), (2) hepatitis B/hepatitis C infection (*N* = 127), (3) more than two drinks per day in the past year (*N* = 1,069) (4) incomplete body measurements index results (*N* = 186); and (5) other necessary covariates (*N* = 5). Finally, there were 3,732 participants in this study (Figure 1).

### Definition and assessment of definition and evaluation of WHR and NALFD

#### WHR

Waist and hip circumferences were measured for each participant by professionally trained health technicians at the Mobile Examination Center (MEC). The examination rooms at the MEC were uniformly equipped to minimize potential errors. These measurements were then utilized to calculate the WHR. The WHR is calculated by dividing the waist circumference by the hip circumference.

#### NAFLD

The gold standard for NAFLD diagnosis is a liver biopsy. However, this method is invasive and is often replaced in the clinic using imaging methods, commonly ultrasound and vibration-controlled transient elastography of the liver. In 2017–2018, NHANES utilized the FibroScan® Touch device to conduct vibration-controlled transient elastography assessments on participants. CAP was used as an indicator of hepatic fat levels, capable of detecting liver fat infiltration exceeding 5%. The NAFLD status criterion employed was CAP = 274 dB/M, a threshold that had been demonstrated to possess 90% sensitivity with 60% specificity. This criterion was widely used to assess hepatic steatosis status and criteria in the US population (19).

### Definition and assessment of other relevant variables

In order to assess the demographic characteristics of people with NAFLD and to reduce possible confounding effects, other variables related to NAFLD were included in this study: (1) Demographic characteristics: gender, age, race, education and BMI (2) Liver function: Alanine aminotransferase (ALT), Aspartate aminotransferase (AST) (3) Hypertension: Participants with three resting blood pressure measurements on different days and three consecutive days of systolic blood pressure ≥ 140 mmHg or diastolic blood pressure ≥ 90 mmHg were defined as hypertensive. (4) An individual diagnosed with diabetes mellitus who meets one or more of the following criteria: (1) Participants with a glycated hemoglobin level greater than 6.5%. (2) Fasting blood glucose level of 7.0 mmol/L. (3) Hyperlipidemia was defined as low-density lipoprotein (LDL) ≥ 130 mg/dL, total cholesterol (TC) ≥ 200 mg/dL, triglycerides (TG) ≥ 150 mg/dL, or high-density lipoprotein (HDL) ≤ 50 mg/dL in females and ≤ 40 mg/dL in males according to the National Cholesterol Education Program (20).

### MR analysis

The data on genetic variants strongly associated with WHR was obtained from the GIANT consortium. They conducted a genome-wide association meta-analysis of WHR by adjusting for BMI. The study recruited 116,740 samples and 2,467,779 SNPs from populations across Europe (21). Non-alcoholic fatty liver data from a study conducted in 2021 with 4,761 NAFLD patients compared to 373,227 controls, analyzing 9,097,254 SNPs (22). These GWAS data could be downloaded from the Integrated Epidemiology Unit’s (IEU) OpenGWAS database.^2^ All subjects provided informed consent, which was reviewed and approved by the local institutional review board.

To identify genetic variants that have a causal effect between exposure (WHR) and outcome (NAFLD), we screened the SNPs obtained. Firstly, we confirmed the genetic variants that were strongly associated with exposure at a genome-wide significance level of *p* < 5^10^−8^. Secondly, we removed SNPs with linkage disequilibrium of r^2^ < 0.001 and kb = 10,000. Finally, we extracted 34 SNPs strongly associated with WHR. Details about data downloading and screening were presented in Table 1.

**Table 1:** Characteristics of GWAS enrolled in the MR study.

Items	GWAS ID	Sample size	Number of SNPs	Population	Download link
WHR	ieu-a-81	116,740	2,467,779	Europeans	https://gwas.mrcieu.ac.uk/
NAFLD	ebi-a-GCST90054782	377,998	9,097,254	Europeans	https://gwas.mrcieu.ac.uk/

NAFLD, Non-alcoholic fatty liver disease; WHR, waist-to-hip ratio; GWAS, Genome-wide association study; SNPs, Single nucleotide polymorphisms.

### Statistical analysis

When analyzing NHANES data, multivariable-adjusted logistic regression models were employed to assess the relationship between WHR and NAFLD. To further examine the correlation between WHR and NAFLD, the WHR was divided into four equal segments. The first quartile (Q1) was employed as the reference value for the purpose of analyzing whether there was variability in the correlation between each interval and NAFLD. The study adjusted for variables that exhibited significant effects on NAFLD in clinical research. Three models were established: Model 1 remained unadjusted; Model 2 was adjusted for gender, age, and BMI; and Model 3 was adjusted for ALT, AST, hypertension, hyperlipidemia and diabetes mellitus, which were incorporated based on Model 2. Results were expressed as odds ratios (OR) or *β* coefficients with 95% confidence intervals [CI]. Subgroups based on gender, age, and BMI were created using Model 3 to facilitate a more detailed examination of the correlation between WHR and NAFLD across diverse demographic categories. Based on the multiple logistic regression model 3, we divided the population into subgroups according to sex, BMI, and age for interaction test analysis. Among them, BMI <25 kg/m^2^ was shown as normal group, 25 kg/m^2^ ≤ BMI <30 kg/m^2^ as overweight group, and BMI ≥30 kg/m^2^ as obese group. Additionally, restricted spline regression (RCS) was employed to examine non-linearity in the WHR-NAFLD association. Given that NHANES data were collected using complex multistage probability sampling, appropriate weights were applied in the statistical analysis of this study.

To investigate the causal relationship between WHR and NAFLD, we conducted a Two-sample MR analysis. We used inverse variance weighting (IVW) as the primary method to evaluate the relationship between exposures and outcomes. To validate the IVW results, four additional MR analysis methods were introduced: MR Egger, Weighted median, simple mode, and weighted mode. The potential heterogeneity was assessed using Cochrane’s Q-test (*p* < 0.5was considered indicative of potential heterogeneity.). The horizontal multiplicity of genetic variation was estimated using MR-Egger intercept (*p* < 0.5 was considered indicative of potential horizontal pleiotropy). Additionally, a leave-one-out analysis was performed to determine that individual SNPs do not contribute significantly to the outcome.

The paper utilized R software version 4.3.2 (R Foundation, Vienna, Austria) and EmpowerStats software (X&Y Solutions Inc., Boston, MA, USA) for statistical analysis.

## Results

### Baseline characteristics of study participants

Participants were categorized into 2,102 Non-NAFLD patients and 1,630 NAFLD patients based on the CAP cutoff of 274 dB/m. The NAFLD patients tended to be older and predominantly male non-Hispanic whites. Additionally, as expected, patients with NAFLD exhibited higher liver inflammation indices and an increased risk of hypertension and diabetes compared to those without NAFLD (Table 2).

**Table 2:** Baseline characteristics of participants.

	Non-NAFLD	NAFLD	*p*-value
Age (years)	46.267 (44.671,47.862)	53.317 (52.316,54.317)	<0.0001
BMI (kg/m^2^)	26.502 (26.015,26.989)	33.454 (32.627,34.281)	<0.0001
Alt (U/L)	18.789 (18.240,19.337)	24.991 (24.090,25.892)	<0.0001
Ast (U/L)	20.553 (19.927,21.180)	21.870 (21.326,22.415)	0.0111
HDL (mg/dL)	57.043 (56.205,57.881)	48.593 (47.630,49.557)	<0.0001
TG (mg/dL)	92.148 (88.943,95.354)	143.124 (128.885,157.363)	<0.0001
TC (mg/dL)	111.243 (108.325,114.162)	112.381 (105.768,118.994)	0.7463
LDL (mg/dL)	188.295 (185.144,191.446)	190.311 (185.680,194.943)	0.2933
WHR (cm)	0.899 (0.891,0.906)	0.969 (0.962,0.976)	<0.0001
Gender (%)			<0.0001
Male	38.785 (36.754,40.855)	49.998 (46.310,53.686)	
Female	61.215 (59.145,63.246)	50.002 (46.314,53.690)	
Race (%)			0.0010
Mexican American	5.985 (3.872,9.144)	9.559 (6.233,14.385)	
Other Hispanic	7.098 (5.417,9.250)	5.972 (4.405,8.049)	
Non-Hispanic White	62.642 (56.159,68.699)	63.528 (57.085,69.521)	
Non-Hispanic Black	13.155 (9.818,17.407)	9.802 (6.919,13.708)	
Other Race	11.120 (7.913,15.408)	11.139 (8.027,15.258)	
Education (%)			0.0306
Less than 9th grade	3.291 (2.411,4.478)	3.785 (2.529,5.629)	
9-11th grade	7.095 (5.592,8.962)	6.401 (5.000,8.161)	
High school graduate/GED or equivalent	24.559 (20.268,29.423)	27.012 (23.312,31.061)	
Some college or AA degree	27.503 (23.746,31.608)	32.425 (28.115,37.055)	
College graduate or above	37.481 (30.912,44.547)	30.257 (24.166,37.131)	
Refused	0.021 (0.002,0.170)	0.016 (0.002,0.132)	
Do not Know	0.050 (0.006,0.441)	0.105 (0.038,0.295)	
Hypertension (%)			0.0002
Non Hypertension	89.318 (87.365,91.000)	84.072 (81.358,86.457)	
Hypertension	10.682 (9.000,12.635)	15.928 (13.543,18.642)	
Diabetes (%)			<0.0001
Non Diabetes	94.924 (93.362,96.134)	76.933 (73.830,79.769)	
Diabetes	5.076 (3.866,6.638)	23.067 (20.231,26.170)	

Data were presented as median (interquartile range) or *n* (%). Mann–Whitney U test for continuous variables. NAFLD, Non-alcoholic fatty liver disease; BMI, Body mass index; WHR, Waist-to-Hip Ratio; ALT, Alanine aminotransferase; AST, Aspartate transaminase.

### Associations between WHR and NAFLD in NHANES

**Figure 2:**
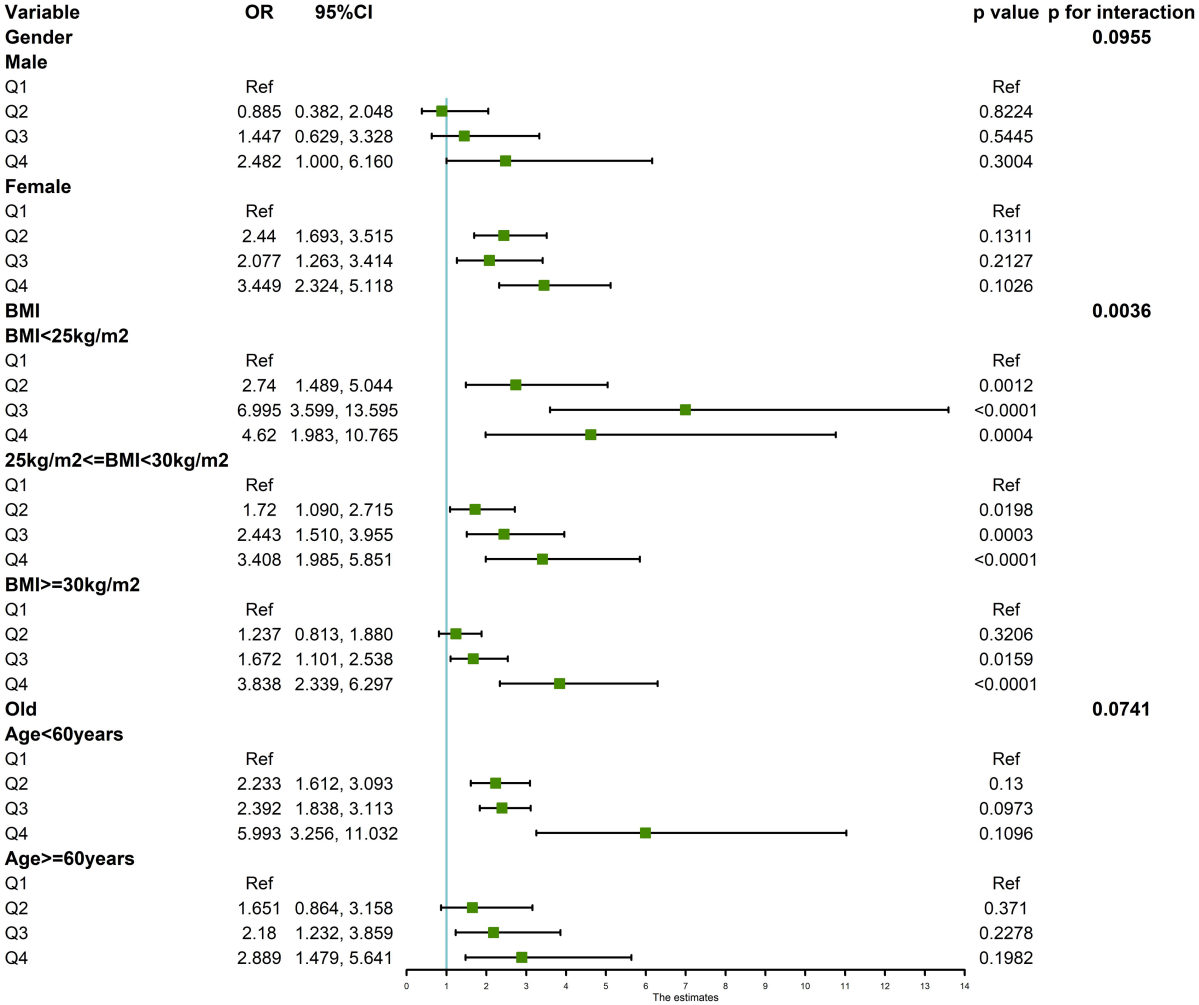
Subgroup analysis of WHR versus NAFLD.

**Figure 3:**
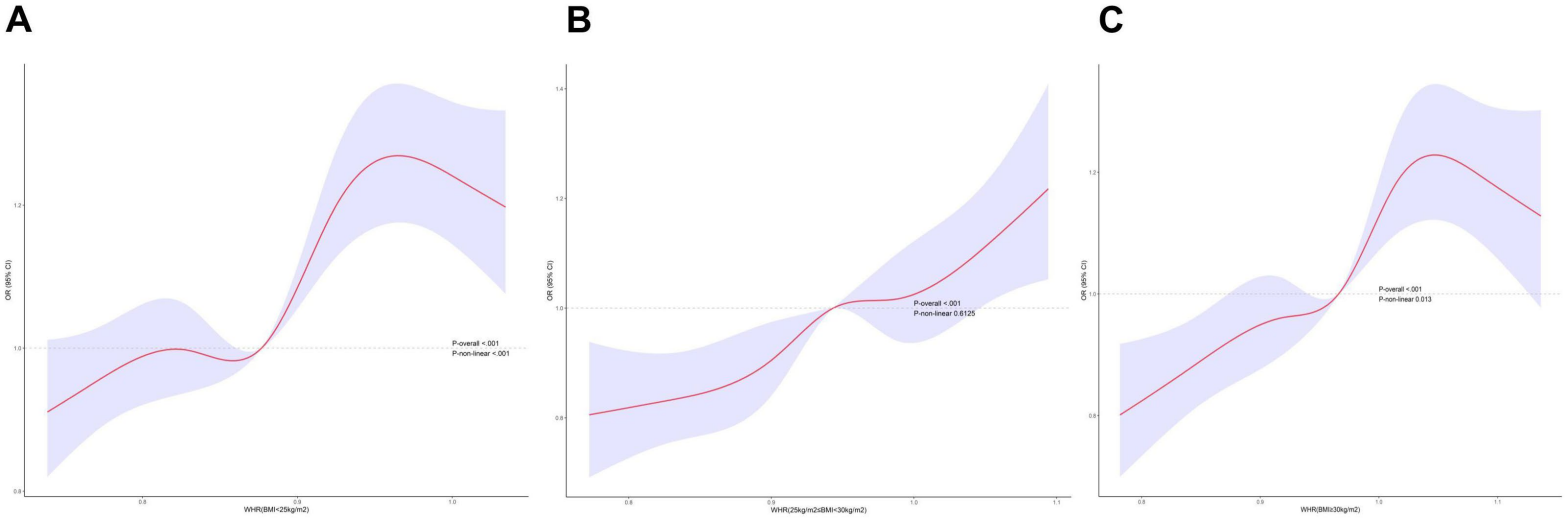
RCS curves of WHR versus NAFLD at different BMIs.

**Figure 4:**
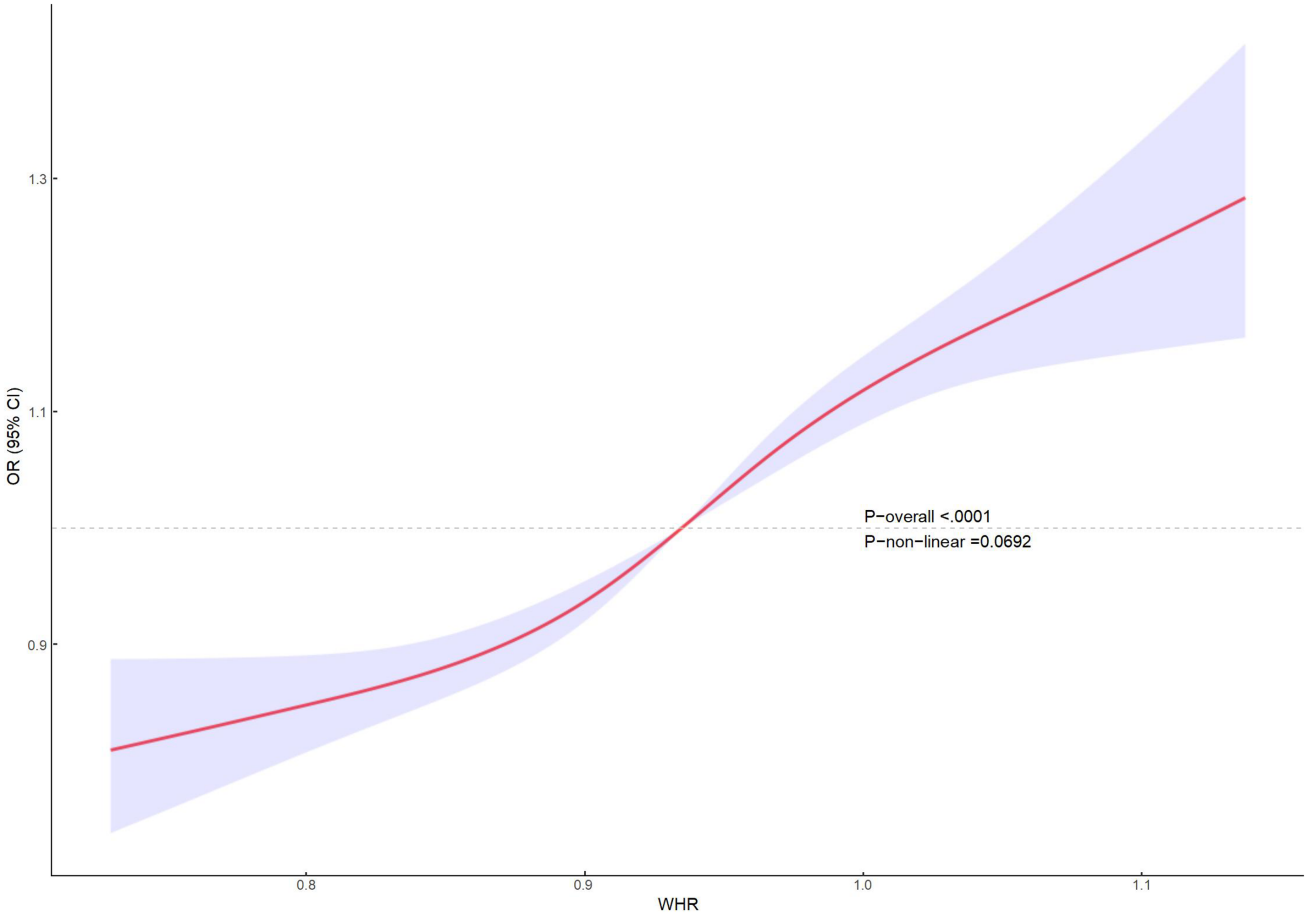
RCS curves of WHR versus NAFLD for all populations.

Multifactorial regression analysis showed a significant association between WHR and NAFLD (Table 3). Unadjusted model 1 showed (OR = 12.85 [95% CI: 9.02, 18.30], *p* < 0.001). To reduce the negative impact of confounders on the results, we adjusted for potential confounders in model 2 (OR = 4.37 [95% CI, 2.90, 6.57], *p* < 0.001) and in the fully adjusted model 3 (OR = 3.70 [95% CI, 2.13, 6.43], *p* = 0.010). The results of our study showed a significant association between WHR and NAFLD even with these adjustments. In the trend regression test, all three models showed a significant correlation (*p* < 0.05). The subgroup analysis showed that WHR was found to be positively associated with the prevalence of NAFLD, irrespective of whether the patients were of a normal or elevated BMI (*p* for interaction<0.05) (Figure 2). The correlation between WHR and NAFLD was more pronounced when the body mass index was lower. Interestingly, when a person’s BMI was in the normal weight range, we found that the Q3 group had about a 2-fold higher correlation with NAFLD compared to the Q4 group. Therefore, we analyzed the BMI subgroups separately for RCS curves. The results suggested a nonlinear relationship between those with BMI <25 kg/m^2^ and those with BMI ≥30 kg/m^2^ (*p* < 0.05), and no nonlinear relationship in the overweight population (*p* > 0.05) (Figure 3). The gender subgroup and age groups demonstrated no statistically significant correlation (*p* for interaction>0.05). The RCS curves suggested that there was no nonlinear relationship between WHR and NAFLD in all participants (*p* > 0.05) (Figure 4). The results of all the above studies showed that the risk of NAFLD tended to increase significantly with increasing WHR.

**Table 3:** Weighted logistic regression analysis models showing the associations between WHR and NAFLD.

Items	Model 1		Model 2		Model 3	
	OR (95%CI)	*p*-value	OR (95%CI)	*p*-value	OR (95%CI)	*p*-value
WHR						
Q1	Ref.	Ref.	Ref.	Ref.	Ref.	Ref.
Q2	3.05 (2.30,4.05)	<0.001	2.07 (1.62,2.65)	<0.001	1.94 (1.55,2.44)	0.005
Q3	5.18 (3.89,6.89)	<0.001	2.47 (1.89,3.24)	<0.001	2.08 (1.51,2.85)	0.011
Q4	12.85 (9.02,18.30)	<0.001	4.37 (2.90,6.57)	<0.001	3.70 (2.13,6.43)	0.010
*p* for trend		<0.001		<0.001		0.007

Data are expressed as OR [95% CI]. Model 1 was unadjusted; Model 2 was adjusted for gender, age, and BMI; Model 3 was adjusted for gender, age, BMI, ALT, AST, hyperlipidemia, hypertension, and diabetes. OR, odds ratio; CI, confidence interval; WHR, waist-to-hip; Q1, 1st quartile; Q2, 2nd quartile; Q3, 3rd quartile; Q4, 4th quartile. NAFLD, Non-alcoholic fatty liver disease; BMI, Body mass index; WHR, Waist-to-Hip Ratio; ALT, Alanine aminotransferase; AST, Aspartate transaminase.

### Causal connections between WHR and the risk of NAFLD in MR

**Figure 5:**
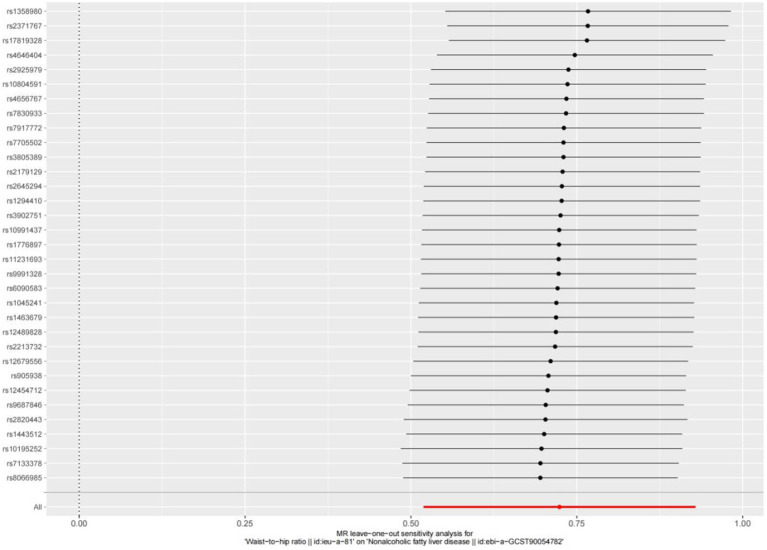
Leave-one-out cross validation.

**Figure 6:**
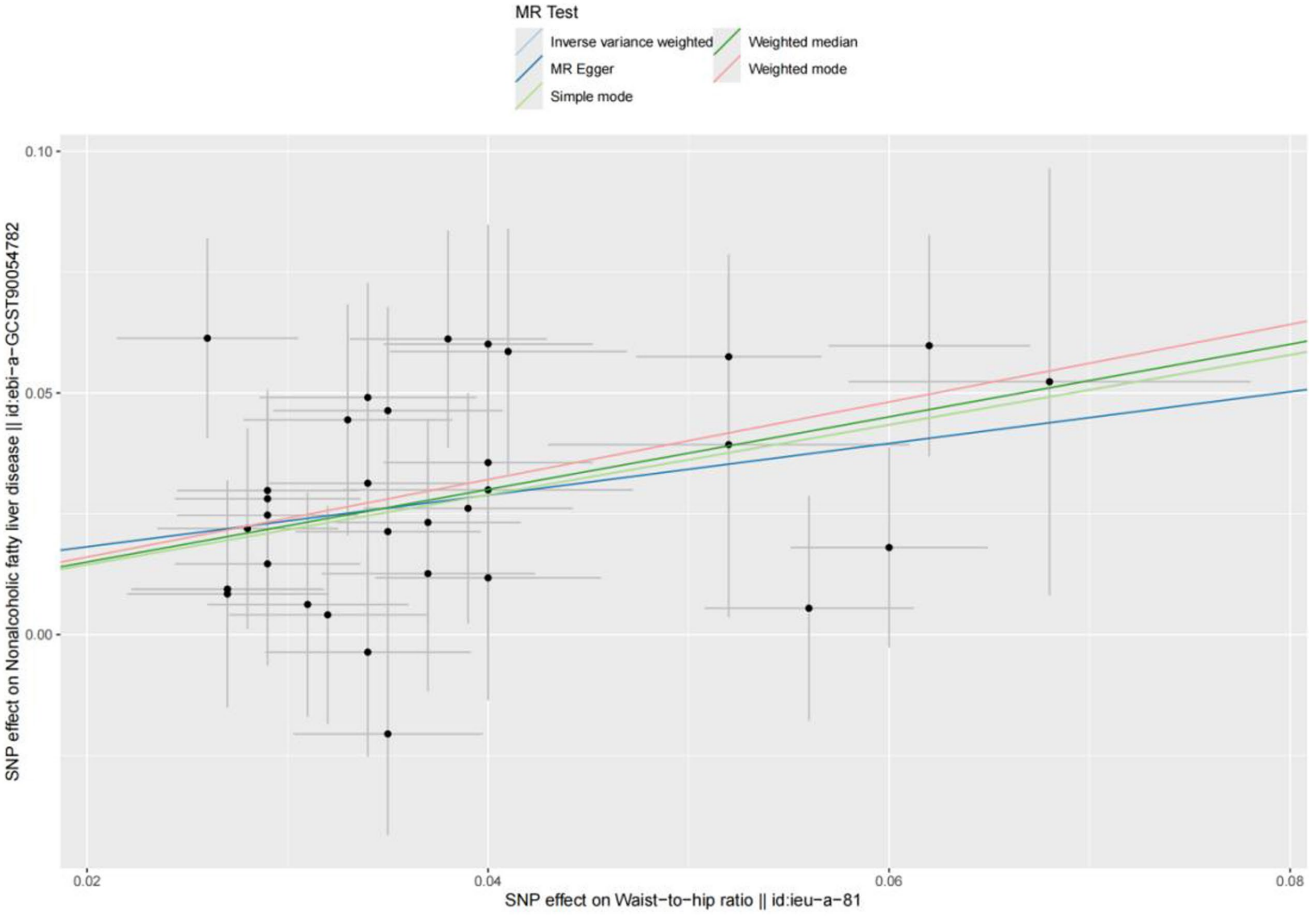
Scatter plot.

Based on cross-sectional data from the NHANES database, a strong correlation was found between WHR and NAFLD. To investigate the causal relationship, a two-sample MR analysis was conducted. In the MR analysis where WHR served as the exposure and NAFLD as the outcome, identified one palindromic SNP (rs1936807), which was subsequently excluded to ensure data reliability. A total of 33 SNPs were included in the study. During MR analysis, the WHR derived from the IVW method exhibited a robust correlation with NAFLD (OR = 2.062 [95% CI: 1.680, 2.531]), consistent with findings from other complementary MR analysis techniques (Table 4). The potential confounding effects of WHR and NAFLD were analyzed. The MR-Egger test showed no polynomial effect (*p* > 0.5), and MR-PRESSO did not detect a horizontal polynomial effect or any abnormal SNPs (*p* > 0.5). Leave-one-out test analysis showed that the causal relationship between WHR and NAFLD was not driven by any single SNP (Figure 5). Furthermore, the symmetry observed in the funnel plot affirms the stability and reliability of our findings (Supplementary Figure S1). Scatterplot Illustrating the Estimated Impact of WHR on NAFLD Through Various MR Analyses (Figure 6).

**Table 4:** MR analysis assessing the causal impact of WHR on NAFLD Risk.

Exposure	MR method	*β*	SE	OR (95% CI)	*p*-value
WHR	MR Egger	0.53	0.4	1.707(0.780,3.734)	0.191
	Weighted median	0.75	0.15	2.120 (1.580,2.842)	<0.001
	IVW	0.72	0.1	2.062 (1.680,2.531)	<0.001
	Simple mode	0.72	0.32	2.061 (1.105,3.841)	0.03
	Weighted mode	0.80	0.32	2.230 (1.189,4.183)	0.018

CI, Confidence interval; IVW, Inverse variance weighted; MR, Mendelian randomization; NAFLD, Non-alcoholic fatty liver disease; WHR, Waist-to-Hip Ratio; OR, Odds ratio; SE, Standard error.

## Discussion

In this study, we employed a cross-sectional analysis of the nationally representative NHANES 2017–2018 cohort in conjunction with a dual-sample MR analysis to investigate the relationship between WHR and NAFLD.

Our results suggested that individuals with higher WHR had a higher risk of developing NAFLD. In subgroup analyses, the correlation between WHR and NAFLD held true for patients with normal BMI, which indicated that WHR was a better surrogate for obesity indicators than BMI. The association between increased WHR and NAFLD was more significant when BMI was lower. In addition, we found a nonlinear relationship between WHR and NAFLD in normal-weight and obese individuals. When BMI was <25 kg/m^2^, we identified an inverted U-shaped correlation, which was consistent with the higher risk of NAFLD in group Q3 compared to group Q4 in the subgroup analysis. In the obese weight group, the correlation with NAFLD was nearly the same in the Q2 and Q3 groups, which was also explained by the nonlinear relationship. The two-sample MR approach also corroborated our results, confirming the causal effect of WHR on NAFLD. Consequently, WHR is a reliable predictor of NAFLD. Numerous prior studies have investigated the association between WHR and NAFLD. This paper represents the first comprehensive analysis to utilize a combination of cross-sectional studies and two-sample MR to examine the relationship between WHR and NAFLD causality.

NAFLD has become one of the most common liver diseases in the world (23). In 2019 statistics alone, there were 1.2 billion cases of NAFLD diagnosed worldwide, with a whopping 168,969 deaths (24). Therefore, it is crucial to enhance our capacity to identify individuals at risk of developing NAFLD, as this will facilitate the prevention of its onset and progression. Obesity is an independent risk factor for NAFLD, potentially due to the close association between obesity and the expansion of adipose tissue, which impairs its capacity to store excess energy, thereby inducing adipocyte dysfunction (25).

Currently, the BMI index is widely utilized for assessing obesity in clinical and epidemiological studies and is also recognized as a risk factor for NAFLD. However, it has been noted that the distribution of fat across different body sites may hold more significance than the overall fat content of the body (26). However, BMI is unable to quantify the amount and distribution of visceral fat, thus appearing as an insufficient indicator of obesity (27). To better elucidate the association between obesity and NAFLD, this paper introduces WHR, a pivotal anthropometric measure of obesity in humans that provides a more nuanced reflection of fat distribution (28, 29). WHR is regarded as an indicator of central obesity and has demonstrated a stronger correlation with clinical outcomes compared to BMI (30). WHR, a prominent marker of steatosis, serves as an indirect anthropometric indicator of body fat distribution and is typically associated with visceral fat volume (31). The potential pathway for visceral fat to impact the liver involves direct drainage into the portal venous system, leading to an excess of free fatty acids exposure in the liver. These fatty acids can be oxidized or converted into triglycerides in the presence of other factors, subsequently being stored in the liver or released into circulation (32). The accumulation of these substances in the liver can result in hepatic fat accumulation, ultimately leading to the development of NAFLD. WHR is also recognized as a measure to evaluate central obesity and its association with complications (33, 34). It has been observed that central obesity is considered one of the most significant predictors of NAFLD in healthy populations. Furthermore, numerous studies have demonstrated that increased abdominal fat in individuals serves as an independent predictor of hepatocellular steatosis, carrying significant implications for the pathogenesis of NAFLD (35, 36). Crucially, WHR has demonstrated a significant association with the onset of NAFLD (37, 38), aligning with our own findings. The aforementioned evidence underscores WHR as a reliable parameter for predicting NAFLD (39).

The results of the nationally representative cross-sectional study conducted in this paper also indicate a strong association between WHR and the occurrence of NAFLD. To further investigate this association, we conducted a two-sample MR approach, which demonstrated a causal effect of WHR on NAFLD. Additionally, sensitivity and other methods were employed to assess the robustness and reliability of our results. This evidence suggests that WHR can be a valuable predictor when assessing the risk of NAFLD and its associated outcomes.

The primary strength of our study lies in its utilization of samples drawn from the nationally representative NHANES database for the cross-sectional analysis, thereby ensuring the high informativeness of our data. Moreover, we adopted a two-sample MR approach to mitigate the inherent challenges of reverse causality and confounding factors often encountered in cross-sectional studies, thus enabling robust causal inference (40). Interestingly, both methodologies employed throughout our study yielded concordant results, affirming that elevated WHR significantly heightens the risk of developing NAFLD. This convergence enhances the credibility and reliability of our findings.

However, it should be noted that our study has certain limitations. Firstly, the diagnosis of NAFLD relied on transient elastography, a highly sensitive yet non-invasive technique. Although it has become an important tool for estimating hepatic fat accumulation (41), the CAP used in this method can be influenced by factors such as BMI, visceral fat content, and intercostal space width. These factors may lead to an underestimation of NAFLD prevalence (42). Secondly, despite our efforts to control for confounders primarily associated with NAFLD in our cross-sectional analysis, there may still be other confounding factors influencing the results. Thirdly, the absence of exploration in our study regarding how WHR affects the pathomechanisms of NAFLD warrants further consideration. Finally, our study focused on European and American populations, which limits the generalizability of our findings to other ethnic groups.

## Conclusion

In conclusion, our study reveals that WHR serves as an indicator of heightened NAFLD risk, as evidenced by cross-sectional and two-sample MR analyses. Given the affordability and non-invasiveness of WHR measurement, this underscores its significance for early mass screening of populations vulnerable to NAFLD.

## Data Availability

The datasets presented in this study can be found in online repositories. The names of the repository/repositories and accession number(s) can be found in the article/Supplementary material.

## Footnotes

^1^https://www.cdc.gov/nchs/nhanes/index.htm

^2^https://gwas.Mrcieu.ac.uk/
