# Supplementary figures and images for: Waist-to-hip ratio and nonalcoholic fatty liver disease: a clinical observational and Mendelian randomization analysis
Source: Front Nutr. 2024 Nov 1;11:1426749. doi: 10.3389/fnut.2024.1426749 (PMC11563977; doi:10.3389/fnut.2024.1426749)

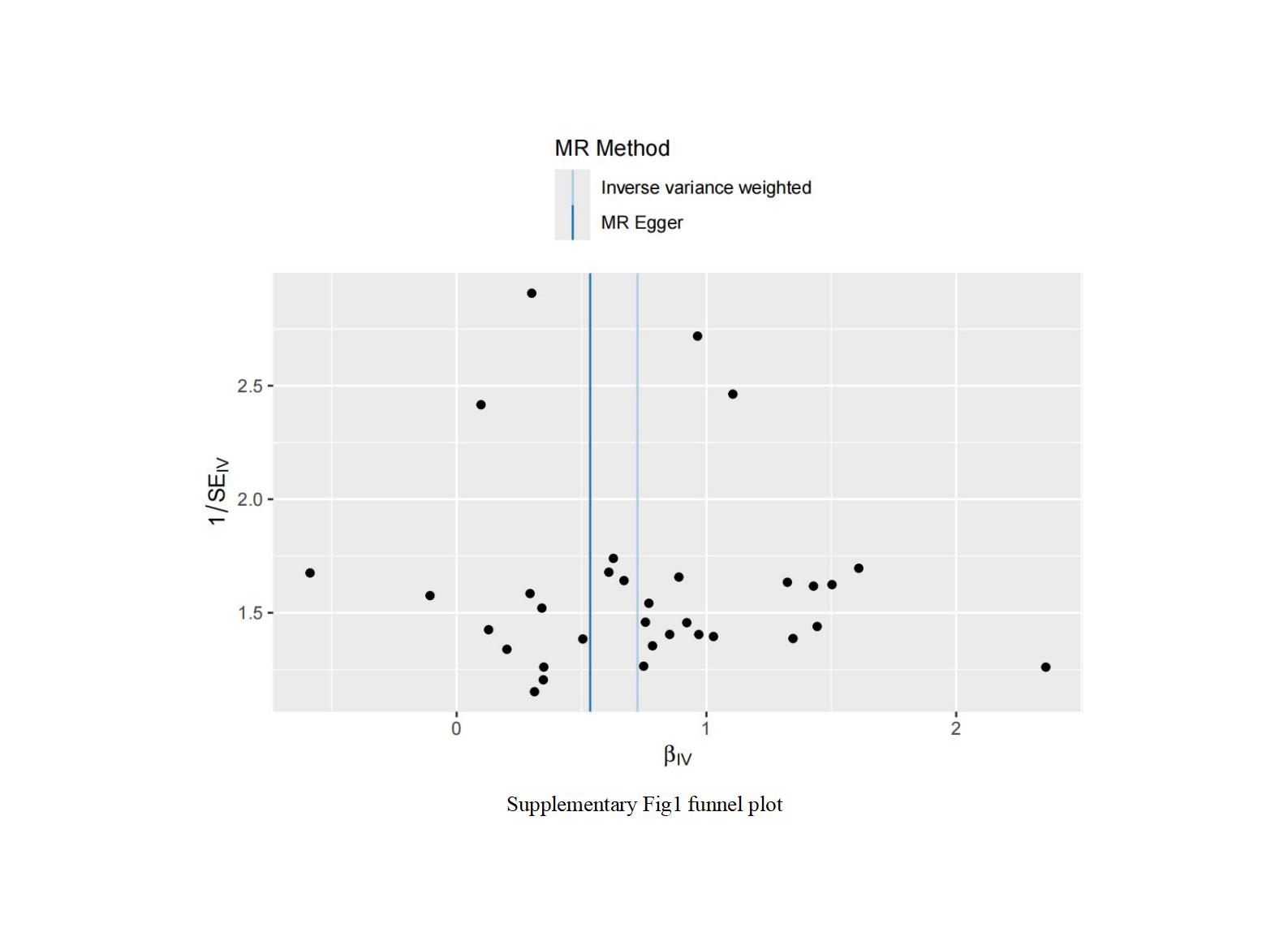

Supplement: Supplementary file 1 [file Image_1.jpeg]
