# Supplementary material for: Waist-to-hip ratio and nonalcoholic fatty liver disease: a clinical observational and Mendelian randomization analysis
Source: Front Nutr. 2024 Nov 1;11:1426749. doi: 10.3389/fnut.2024.1426749 (PMC11563977; doi:10.3389/fnut.2024.1426749)
Supplement: Supplementary file 3 [file Table_2.docx]

**STROBE-MR checklist of recommended items to address in reports of Mendelian randomization studies**^1^ ^2^

| **Item No.** | **Section** | **Checklist item** | **Page No.** | **Relevant text from manuscript** |
| --- | --- | --- | --- | --- |
| 1 | **TITLE and ABSTRACT** | Indicate Mendelian randomization (MR) as the study’s design in the title and/or the abstract if that is a main purpose of the study |  | Waist-to-Hip Ratio and Nonalcoholic Fatty Liver Disease: A Clinical Observational and Mendelian Randomization Analysis. |
|  | **INTRODUCTION** |  |  |  |
| 2 | **Background** | Explain the scientific background and rationale for the reported study. What is the exposure? Is a potential causal relationship between exposure and outcome plausible? Justify why MR is a helpful method to address the study question |  | NAFLD is a metabolic stress liver injury caused by abnormal accumulation of lipids in hepatocytes and is gradually replacing viral hepatitis as the most common chronic liver disease[7]. Without timely intervention and treatment, the disease can progress from simple steatosis (NAFL) to nonalcoholic steatohepatitis (NASH) and even to irreversible cirrhosis and hepatocellular carcinoma (HCC). NAFLD is also closely related to the development of cardiovascular diseases, diabetic complications, chronic kidney disease, and malignancy[8-10]. Therefore, it is of utmost importance to prevent and treat NAFLD as early as possible to reduce its progression to adverse outcomes.  Obesity is one of the independent risk factors for NAFLD. A meta-analysis demonstrated that obese individuals have a 3.5-fold increased risk of NAFLD, and there is a significant dose-dependent relationship between obesity and NAFLD risk[11]. A variety of human obesity measures have been employed to assess the degree of risk for metabolic diseases in humans, including BMI, waist circumference (WC), waist-to-height ratio (WHtR), and other anthropometric measures. The current common metrics for determining obesity are the BMI. A study by Younossi ZM[12]. demonstrated that the prevalence of NAFLD increased with BMI. Nevertheless, an increasing number of studies have demonstrated that NAFLD can also occur in individuals with a normal BMI and an excessive abdominal circumference, or what is commonly referred to as "thin individuals with NAFLD." A meta-analysis conducted by Tang[13] revealed that thin individuals with NAFLD accounted for approximately 13.11% of the global population, with a prevalence as high as 14.55% in the Asian region. This indicates that the use of BMI alone to diagnose obesity is erroneous. One possible explanation for this is that BMI does not consider the distribution of adipose tissue, particularly in the trunk region[14] .  Nevertheless, abdominal obesity and NAFLD frequently co-occur in the same individual, forming a complex, bidirectional relationship. Existing studies have various limitations, including insufficient sample sizes and cross-sectional designs that are susceptible to confounding factors, making it challenging to ascertain causality.MR has emerged as a valuable complement to cross-sectional observational studies in assessing the causal effect of a specified risk factor on an outcome at the genetic level[17]. |
| 3 | **Objectives** | State specific objectives clearly, including pre-specified causal hypotheses (if any). State that MR is a method that, under specific assumptions, intends to estimate causal effects |  | MR has emerged as a valuable complement to cross-sectional observational studies in assessing the causal effect of a specified risk factor on an outcome at the genetic level[17]. The advantage of MR is that genetic variants are established before birth and randomly assigned at meiosis and fertilization, which greatly reduces the influence of other factors on the outcome and addresses the issue of avoiding the association between exposure and reverse causation of the outcome[18]. Consequently, we employed two-sample MR analysis to supplement cross-sectional studies and comprehensively evaluate the relationship. |
|  | **METHODS** |  |  |  |
| 4 | **Study design and data sources** | Present key elements of the study design early in the article. Consider including a table listing sources of data for all phases of the study. For each data source contributing to the analysis, describe the following: |  |  |
|  | a) | Setting: Describe the study design and the underlying population, if possible. Describe the setting, locations, and relevant dates, including periods of recruitment, exposure, follow-up, and data collection, when available. |  | The data on genetic variants strongly associated with WHR was obtained from the GIANT consortium. They conducted a genome-wide association meta-analysis of WHR by adjusting for BMI. The study recruited 116,740 samples and 2,467,779 SNPs from populations across Europe[21]. Non-alcoholic fatty liver data from a study conducted in 2021 with 4,761 NAFLD patients compared to 373,227 controls, analyzing 9,097,254 SNPs[22].These GWAS data could be downloaded from the Integrated Epidemiology Unit's (IEU) OpenGWAS database (https://gwas.mrcieu.ac.uk/). All subjects provided informed consent, which was reviewed and approved by the local institutional review board. |
|  | b) | Participants: Give the eligibility criteria, and the sources and methods of selection of participants. Report the sample size, and whether any power or sample size calculations were carried out prior to the main analysis |  | To identify genetic variants that have a causal effect between exposure (WHR) and outcome (NAFLD), we screened the SNPs obtained. Firstly, we confirmed the genetic variants that were strongly associated with exposure at a genome-wide significance level of p<5^10-8. Secondly, we removed SNPs with linkage disequilibrium of r2<0.001 and kb=10,000. Finally, we extracted 34 SNPs strongly associated with WHR. Details about data downloading and screening were presented in Table 1. |
|  | c) | Describe measurement, quality control and selection of genetic variants |  | Firstly, we confirmed the genetic variants that were strongly associated with exposure at a genome-wide significance level of p<5^10-8. Secondly, we removed SNPs with linkage disequilibrium of r2<0.001 and kb=10,000. Finally, we extracted 34 SNPs strongly associated with WHR. Details about data downloading and screening were presented in Table 1. |
|  | d) | For each exposure, outcome, and other relevant variables, describe methods of assessment and diagnostic criteria for diseases |  | To investigate the causal relationship between WHR and NAFLD, we conducted a Two-sample MR analysis. We used inverse variance weighting (IVW) as the primary method to evaluate the relationship between exposures and outcomes. To validate the IVW results, four additional MR analysis methods were introduced: MR Egger, Weighted median, Simple mode, and Weighted mode. The potential heterogeneity was assessed using Cochrane's Q-test (p<0.5was considered indicative of potential heterogeneity.). The horizontal multiplicity of genetic variation was estimated using MR-Egger intercept (p<0.5 was considered indicative of potential horizontal pleiotropy). Additionally, a leave-one-out analysis was performed to determine that individual SNPs do not contribute significantly to the outcome. |
|  | e) | Provide details of ethics committee approval and participant informed consent, if relevant |  | These GWAS data could be downloaded from the Integrated Epidemiology Unit's (IEU) OpenGWAS database (https://gwas.mrcieu.ac.uk/). All subjects provided informed consent, which was reviewed and approved by the local institutional review board. |
| 5 | **Assumptions** | Explicitly state the three core IV assumptions for the main analysis (relevance, independence and exclusion restriction) as well assumptions for any additional or sensitivity analysis |  | To investigate the causal relationship between WHR and NAFLD, we conducted a Two-sample MR analysis. We used inverse variance weighting (IVW) as the primary method to evaluate the relationship between exposures and outcomes. To validate the IVW results, four additional MR analysis methods were introduced: MR Egger, Weighted median, Simple mode, and Weighted mode. The potential heterogeneity was assessed using Cochrane's Q-test (p<0.5was considered indicative of potential heterogeneity.). The horizontal multiplicity of genetic variation was estimated using MR-Egger intercept (p<0.5 was considered indicative of potential horizontal pleiotropy). Additionally, a leave-one-out analysis was performed to determine that individual SNPs do not contribute significantly to the outcome. |
| 6 | **Statistical methods: main analysis** | Describe statistical methods and statistics used |  |  |
|  | a) | Describe how quantitative variables were handled in the analyses (i.e., scale, units, model) |  | To investigate the causal relationship between WHR and NAFLD, we conducted a Two-sample MR analysis. We used inverse variance weighting (IVW) as the primary method to evaluate the relationship between exposures and outcomes. To validate the IVW results, four additional MR analysis methods were introduced: MR Egger, Weighted median, Simple mode, and Weighted mode. The potential heterogeneity was assessed using Cochrane's Q-test (p<0.5was considered indicative of potential heterogeneity.). The horizontal multiplicity of genetic variation was estimated using MR-Egger intercept (p<0.5 was considered indicative of potential horizontal pleiotropy). Additionally, a leave-one-out analysis was performed to determine that individual SNPs do not contribute significantly to the outcome. |
|  | b) | Describe how genetic variants were handled in the analyses and, if applicable, how their weights were selected |  |  |
|  | c) | Describe the MR estimator (e.g. two-stage least squares, Wald ratio) and related statistics. Detail the included covariates and, in case of two-sample MR, whether the same covariate set was used for adjustment in the two samples |  | The data on genetic variants strongly associated with WHR was obtained from the GIANT consortium. They conducted a genome-wide association meta-analysis of WHR by adjusting for BMI. The study recruited 116,740 samples and 2,467,779 SNPs from populations across Europe[21]. Non-alcoholic fatty liver data from a study conducted in 2021 with 4,761 NAFLD patients compared to 373,227 controls, analyzing 9,097,254 SNPs[22] |
|  | d) | Explain how missing data were addressed |  |  |
|  | e) | If applicable, indicate how multiple testing was addressed |  |  |
| 7 | **Assessment of assumptions** | Describe any methods or prior knowledge used to assess the assumptions or justify their validity |  | We used inverse variance weighting (IVW) as the primary method to evaluate the relationship between exposures and outcomes. To validate the IVW results, four additional MR analysis methods were introduced: MR Egger, Weighted median, Simple mode, and Weighted mode. The potential heterogeneity was assessed using Cochrane's Q-test (p<0.5was considered indicative of potential heterogeneity.). The horizontal multiplicity of genetic variation was estimated using MR-Egger intercept (p<0.5 was considered indicative of potential horizontal pleiotropy). Additionally, a leave-one-out analysis was performed to determine that individual SNPs do not contribute significantly to the outcome. |
| 8 | **Sensitivity analyses and additional analyses** | Describe any sensitivity analyses or additional analyses performed (e.g. comparison of effect estimates from different approaches, independent replication, bias analytic techniques, validation of instruments, simulations) |  | The potential heterogeneity was assessed using Cochrane's Q-test (p<0.5was considered indicative of potential heterogeneity.). The horizontal multiplicity of genetic variation was estimated using MR-Egger intercept (p<0.5 was considered indicative of potential horizontal pleiotropy). Additionally, a leave-one-out analysis was performed to determine that individual SNPs do not contribute significantly to the outcome. |
| 9 | **Software and pre-registration** |  |  |  |
|  | a) | Name statistical software and package(s), including version and settings used |  | The paper utilized R software version 4.3.2 (R Foundation, Vienna, Austria) and EmpowerStats software (X&Y Solutions Inc., Boston, MA, USA) for statistical analysis. |
|  | b) | State whether the study protocol and details were pre-registered (as well as when and where) |  | All subjects provided informed consent, which was reviewed and approved by the local institutional review board. |
|  | **RESULTS** |  |  |  |
| 10 | **Descriptive data** |  |  |  |
|  | a) | Report the numbers of individuals at each stage of included studies and reasons for exclusion. Consider use of a flow diagram |  | Fig1 |
|  | b) | Report summary statistics for phenotypic exposure(s), outcome(s), and other relevant variables (e.g. means, SDs, proportions) |  | Table1 |
|  | c) | If the data sources include meta-analyses of previous studies, provide the assessments of heterogeneity across these studies |  |  |
|  | d) | For two-sample MR:  i.  Provide justification of the similarity of the genetic variant-exposure associations between the exposure and outcome samples  ii.  Provide information on the number of individuals who overlap between the exposure and outcome studies |  | MR has emerged as a valuable complement to cross-sectional observational studies in assessing the causal effect of a specified risk factor on an outcome at the genetic level[17]. The advantage of MR is that genetic variants are established before birth and randomly assigned at meiosis and fertilization, which greatly reduces the influence of other factors on the outcome and addresses the issue of avoiding the association between exposure and reverse causation of the outcome[18]. Consequently, we employed two-sample MR analysis to supplement cross-sectional studies and comprehensively evaluate the relationship.  To investigate the causal relationship, a two-sample MR analysis was conducted.In the MR analysis where WHR served as the exposure and NAFLD as the outcome, identified one palindromic SNP (rs1936807), which was subsequently excluded to ensure data reliability. A total of 33 SNPs were included in the study. During MR analysis, the WHR derived from the IVW method exhibited a robust correlation with NAFLD (OR = 2.062 [95% CI: 1.680, 2.531]), consistent with findings from other complementary MR analysis techniques (Table 4).The potential confounding effects of WHR and NAFLD were analyzed. The MR-Egger test showed no polynomial effect (p > 0.5), and MR-PRESSO did not detect a horizontal polynomial effect or any abnormal SNPs (p>0.5). Leave-one-out test analysis showed that the causal relationship between WHR and NAFLD was not driven by any single SNP (Fig.4). Furthermore, the symmetry observed in the funnel plot affirms the stability and reliability of our findings (Supplementary Fig.1).Scatterplot Illustrating the Estimated Impact of WHR on NAFLD Through Various MR Analyses (Fig.5). |
| 11 | **Main results** |  |  |  |
|  | a) | Report the associations between genetic variant and exposure, and between genetic variant and outcome, preferably on an interpretable scale |  | Based on cross-sectional data from the NHANES database, a strong correlation was found between WHR and NAFLD. To investigate the causal relationship, a two-sample MR analysis was conducted.In the MR analysis where WHR served as the exposure and NAFLD as the outcome, identified one palindromic SNP (rs1936807), which was subsequently excluded to ensure data reliability. A total of 33 SNPs were included in the study. |
|  | b) | Report MR estimates of the relationship between exposure and outcome, and the measures of uncertainty from the MR analysis, on an interpretable scale, such as odds ratio or relative risk per SD difference |  | During MR analysis, the WHR derived from the IVW method exhibited a robust correlation with NAFLD (OR = 2.062 [95% CI: 1.680, 2.531]), consistent with findings from other complementary MR analysis techniques (Table 4). |
|  | c) | If relevant, consider translating estimates of relative risk into absolute risk for a meaningful time period |  |  |
|  | d) | Consider plots to visualize results (e.g. forest plot, scatterplot of associations between genetic variants and outcome versus between genetic variants and exposure) |  | Fig3 Fig4 Fig5 |
| 12 | **Assessment of assumptions** |  |  |  |
|  | a) | Report the assessment of the validity of the assumptions |  | The potential confounding effects of WHR and NAFLD were analyzed. The MR-Egger test showed no polynomial effect (p > 0.5), and MR-PRESSO did not detect a horizontal polynomial effect or any abnormal SNPs (p>0.5). Leave-one-out test analysis showed that the causal relationship between WHR and NAFLD was not driven by any single SNP (Fig.4). Furthermore, the symmetry observed in the funnel plot affirms the stability and reliability of our findings (Supplementary Fig.1).Scatterplot Illustrating the Estimated Impact of WHR on NAFLD Through Various MR Analyses (Fig.5). |
|  | b) | Report any additional statistics (e.g., assessments of heterogeneity across genetic variants, such as *I^2^*, Q statistic or E-value) |  | The potential confounding effects of WHR and NAFLD were analyzed. The MR-Egger test showed no polynomial effect (p > 0.5), and MR-PRESSO did not detect a horizontal polynomial effect or any abnormal SNPs (p>0.5). |
| 13 | **Sensitivity analyses and additional analyses** |  |  |  |
|  | a) | Report any sensitivity analyses to assess the robustness of the main results to violations of the assumptions |  | The potential confounding effects of WHR and NAFLD were analyzed. The MR-Egger test showed no polynomial effect (p > 0.5), and MR-PRESSO did not detect a horizontal polynomial effect or any abnormal SNPs (p>0.5). Leave-one-out test analysis showed that the causal relationship between WHR and NAFLD was not driven by any single SNP (Fig.4). Furthermore, the symmetry observed in the funnel plot affirms the stability and reliability of our findings (Supplementary Fig.1).Scatterplot Illustrating the Estimated Impact of WHR on NAFLD Through Various MR Analyses (Fig.5). |
|  | b) | Report results from other sensitivity analyses or additional analyses |  | The potential confounding effects of WHR and NAFLD were analyzed. The MR-Egger test showed no polynomial effect (p > 0.5), and MR-PRESSO did not detect a horizontal polynomial effect or any abnormal SNPs (p>0.5). Leave-one-out test analysis showed that the causal relationship between WHR and NAFLD was not driven by any single SNP (Fig.4). Furthermore, the symmetry observed in the funnel plot affirms the stability and reliability of our findings (Supplementary Fig.1).Scatterplot Illustrating the Estimated Impact of WHR on NAFLD Through Various MR Analyses (Fig.5). |
|  | c) | Report any assessment of direction of causal relationship (e.g., bidirectional MR) |  | During MR analysis, the WHR derived from the IVW method exhibited a robust correlation with NAFLD (OR = 2.062 [95% CI: 1.680, 2.531]), consistent with findings from other complementary MR analysis techniques (Table 4). |
|  | d) | When relevant, report and compare with estimates from non-MR analyses |  |  |
|  | e) | Consider additional plots to visualize results (e.g., leave-one-out analyses) |  | Fig3 Fig4 Fig5 |
|  | **DISCUSSION** |  |  |  |
| 14 | **Key results** | Summarize key results with reference to study objectives |  | The two-sample MR approach also corroborated our results, confirming the causal effect of WHR on NAFLD. Consequently, WHR is a reliable predictor of NAFLD.Numerous prior studies have investigated the association between WHR and NAFLD. This paper represents the first comprehensive analysis to utilize a combination of cross-sectional studies and two-sample MR to examine the relationship between WHR and NAFLD causality. |
| 15 | **Limitations** | Discuss limitations of the study, taking into account the validity of the IV assumptions, other sources of potential bias, and imprecision. Discuss both direction and magnitude of any potential bias and any efforts to address them |  | . Firstly, the diagnosis of NAFLD relied on transient elastography, a highly sensitive yet non-invasive technique. Although it has become an important tool for estimating hepatic fat accumulation[41], the CAP used in this method can be influenced by factors such as BMI, visceral fat content, and intercostal space width. These factors may lead to an underestimation of NAFLD prevalence[42]. Secondly, despite our efforts to control for confounders primarily associated with NAFLD in our cross-sectional analysis, there may still be other confounding factors influencing the results.Thirdly, the absence of exploration in our study regarding how WHR affects the pathomechanisms of NAFLD warrants further consideration. Finally, our study focused on European and American populations, which limits the generalizability of our findings to other ethnic groups. |
| 16 | **Interpretation** |  |  |  |
|  | a) | Meaning: Give a cautious overall interpretation of results in the context of their limitations and in comparison with other studies |  | Interestingly, both methodologies employed throughout our study yielded concordant results, affirming that elevated WHR significantly heightens the risk of developing NAFLD. This convergence enhances the credibility and reliability of our findings. |
|  | b) | Mechanism: Discuss underlying biological mechanisms that could drive a potential causal relationship between the investigated exposure and the outcome, and whether the gene-environment equivalence assumption is reasonable. Use causal language carefully, clarifying that IV estimates may provide causal effects only under certain assumptions |  | Furthermore, numerous studies have demonstrated that increased abdominal fat in individuals serves as an independent predictor of hepatocellular steatosis, carrying significant implications for the pathogenesis of NAFLD[35, 36].Crucially, WHR has demonstrated a significant association with the onset of NAFLD[37, 38], aligning with our own findings. The aforementioned evidence underscores WHR as a reliable parameter for predicting NAFLD[39] |
|  | c) | Clinical relevance: Discuss whether the results have clinical or public policy relevance, and to what extent they inform effect sizes of possible interventions |  | However, it should be noted that our study has certain limitations. Firstly, the diagnosis of NAFLD relied on transient elastography, a highly sensitive yet non-invasive technique. Although it has become an important tool for estimating hepatic fat accumulation[41], the CAP used in this method can be influenced by factors such as BMI, visceral fat content, and intercostal space width. These factors may lead to an underestimation of NAFLD prevalence[42]. Secondly, despite our efforts to control for confounders primarily associated with NAFLD in our cross-sectional analysis, there may still be other confounding factors influencing the results.Thirdly, the absence of exploration in our study regarding how WHR affects the pathomechanisms of NAFLD warrants further consideration. Finally, our study focused on European and American populations, which limits the generalizability of our findings to other ethnic groups. |
| 17 | **Generalizability** | Discuss the generalizability of the study results (a) to other populations, (b) across other exposure periods/timings, and (c) across other levels of exposure |  | The results of the nationally representative cross-sectional study conducted in this paper also indicate a strong association between WHR and the occurrence of NAFLD. To further investigate this association, we conducted a two-sample MR approach, which demonstrated a causal effect of WHR on NAFLD. Additionally, sensitivity and other methods were employed to assess the robustness and reliability of our results. This evidence suggests that WHR can be a valuable predictor when assessing the risk of NAFLD and its associated outcomes. |
|  | **OTHER INFORMATION** |  |  |  |
| 18 | **Funding** | Describe sources of funding and the role of funders in the present study and, if applicable, sources of funding for the databases and original study or studies on which the present study is based |  | The authors affirm that not receive any financial support for conducting the research, contributing to the authorship, or publishing this article. |
| 19 | **Data and data sharing** | Provide the data used to perform all analyses or report where and how the data can be accessed, and reference these sources in the article. Provide the statistical code needed to reproduce the results in the article, or report whether the code is publicly accessible and if so, where |  | These GWAS data could be downloaded from the Integrated Epidemiology Unit's (IEU) OpenGWAS database (https://gwas.mrcieu.ac.uk/).  Table1 |
| 20 | **Conflicts of Interest** | All authors should declare all potential conflicts of interest |  | The authors affirm that the research was carried out without any commercial or financial associations that might pose a conflict of interest. |

This checklist is copyrighted by the Equator Network under the Creative Commons Attribution 3.0 Unported (CC BY 3.0) license.

1. Skrivankova VW, Richmond RC, Woolf BAR, Yarmolinsky J, Davies NM, Swanson SA, et al. Strengthening the Reporting of Observational Studies in Epidemiology using Mendelian Randomization (STROBE-MR) Statement. JAMA. 2021;under review.

2. Skrivankova VW, Richmond RC, Woolf BAR, Davies NM, Swanson SA, VanderWeele TJ, et al. Strengthening the Reporting of Observational Studies in Epidemiology using Mendelian Randomisation (STROBE-MR): Explanation and Elaboration. BMJ. 2021;375:n2233.
